# Negative, Null and Beneficial Effects of Drinking Water on Energy Intake, Energy Expenditure, Fat Oxidation and Weight Change in Randomized Trials: A Qualitative Review

**DOI:** 10.3390/nu8010019

**Published:** 2016-01-02

**Authors:** Jodi J. D. Stookey

**Affiliations:** Children’s Hospital Oakland Research Institute, 5700 Martin Luther King Jr. Way, Oakland, CA 94609, USA; jstookey@chori.org; Tel.: +1-415-312-0237; Fax: +1-415-440-4453

**Keywords:** drinking water, energy intake, fat oxidation, weight loss, weight management

## Abstract

Drinking water has heterogeneous effects on energy intake (EI), energy expenditure (EE), fat oxidation (FO) and weight change in randomized controlled trials (RCTs) involving adults and/or children. The aim of this qualitative review of RCTs was to identify conditions associated with negative, null and beneficial effects of drinking water on EI, EE, FO and weight, to generate hypotheses about ways to optimize drinking water interventions for weight management. RCT conditions that are associated with negative or null effects of drinking water on EI, EE and/or FO in the short term are associated with negative or null effects on weight over the longer term. RCT conditions that are associated with lower EI, increased EE and/or increased FO in the short term are associated with less weight gain or greater weight loss over time. Drinking water instead of caloric beverages decreases EI when food intake is *ad libitum*. Drinking water increases EE in metabolically-inflexible, obese individuals. Drinking water increases FO when blood carbohydrate and/or insulin concentrations are not elevated and when it is consumed instead of caloric beverages or in volumes that alter hydration status. Further research is needed to confirm the observed associations and to determine if/what specific conditions optimize drinking water interventions for weight management.

## 1. Introduction

The CDC, USDA, American Medical Association, American Diabetes Association, American Heart Association and American Academy of Pediatrics recommend drinking water, either instead of other beverages or in greater volume, for weight management (see Table 1). Public health initiatives in the U.S., U.K., Australia, France, Belgium, Spain, Greece, Poland, Mexico, Israel and Taiwan are actively promoting drinking water for weight management [1,2,3,4,5,6,7,8,9,10,11,12,13,14,15,16,17,18].

Despite clear efforts and investment to promote drinking water for weight management, in countries around the world, the scientific community remains uncertain whether water plays a causal role in weight management or merely correlates with other causal factors [19]. Consumers of drinking water differ from non-consumers with respect to several key obesity risk factors, including other beverage intake, diet composition, physical activity, stress and smoking [20]. While some propose that promoting the single behavior of drinking water instead of other beverages may be effective against obesity (e.g., [21,22]), others suggest that a single-component intervention approach may be too simplistic [23].

**Table 1:** Example recommendations to drink water for weight management.

Source	Content	Reference
U.S., Let’s Move!	“Pass on sugar-sweetened drinks and choose water instead. Drink 100% juice without added sugar—fruit juice is sweet and tastes great on its own! Choose 1% or skim milk. Make water exciting! Add a slice of lemon, lime, cucumber or watermelon, or try sparkling water. Add a splash of 100% juice to water or sparkling water. If you’re having soda or juice for a treat, choose the small size.”	[1]
U.S., Drink Up!		[2]
U.S., Centers for Disease Control (CDC)	“Rethink your drink”	[3]
U.S., Department of Agriculture (USDA)	“Make better beverage choices…drink water”	[4]
U.S., American Medical Association	“Drink water instead of sugary drinks”	[5]
U.S., American Diabetes Association	“Drink water—Limit sugar-sweetened drinks including sodas, juices, sports drinks, and coffee drinks. These drinks add calories with little or no nutritional value.”	[6]
U.S., American Heart Association	“Rethink Your Drink. Replace sugary drinks in your diet”	[7]
U.S., American Academy of Pediatrics (AAP)	“Sports drinks contain extra calories that children don’t need, and could contribute to obesity and tooth decay. It’s better for children to drink water during and after exercise”	[8,9]
U.S.: The Weight of the Nation	“Drink water instead of sugary drinks”	[10]
U.S.: “Pouring on the Pounds”	Campaign in the New York City public transport system. “Don’t drink yourself fat. Cut back on soda and other sugary beverages. Go with water, seltzer or low-fat milk instead”. “Your kids could be drinking themselves sick”, “You could be drinking yourself sick”. “Swap sugary drinks for water, fat-free milk and fresh fruit.”	[11]
Poland: “Mum, Dad I prefer water!”	“It has zero calories-it can help maintain proper energy balance.”	[12]
Australia: “Water. The original cool drink.”	“Sweet drinks are an extra food and should only be consumed occasionally. Regular consumption of sweet drinks can cause tooth decay and lead to excess weight gain. Sweet drinks include soft drinks, fruit drinks, cordial, flavored mineral waters, sports waters and sports drinks, energy drinks and fruit juice. Serving water or plain milk at meal times at school camps and events is a great start.”	[13]
Mexico	National campaign to prevent obesity and diabetes includes “funds... to increase access to drinking water in schools.”	[14]
EPODE International Network	Rotterdam Social Marketing Campaign messages include “You are a good and strong mum when you give your children at least twice a day water”, A story about Aquaman, a hero that becomes very strong by drinking water and working out, with pictures of popular football players and popstars.	[15]
U.K.: “Wise up on water!”	“Replacing soft drinks in the diet with water (which has no calories) can help with weight control.” “Children’s water requirements vary with age…”	[16]
Finland	To reduce/prevent obesity, school food policies include: “Drinking water must be provided and must be easily accessible” during school lunch. Water and skimmed/semi-skimmed milk are mentioned as parts of a balanced meal. Products should have a maximum of 1% fat.	[17]
Israel, Finland	Program to reduce obesity in populations affected by diabetes.	[18]
Hungary: Hungarian Aqua Promoting Programme in the Young (HAPPY)	To reduce the excessive consumption of sugary drinks and popularize water consumption among primary school students. Nationwide messages (since 2010) promote water consumption by educating students on adequate fluid consumption and makes free water available on school premises.	[11]
Tonga: Health Promotion Foundation, “A Mouthful of Sugar”	The print campaign features a bottle of soda with the label “diabetes”, and healthier alternatives, such as water or coconut water.	[11]

Reflecting uncertainty about the causal mechanism(s) and factors necessary for effect, recommendations to drink water for weight management are relatively vague. The guidelines in Table 1, for example, lack details and consistency about why drinking water can impact weight management, who should drink water, and when and how to drink water to leverage the causal mechanism. Some guidelines inconsistently recommend drinking water instead of “sugar-sweetened” beverages “to avoid beverage calories”, while at the same time recommending 100% juice, non-fat or low-fat milk, which, like cola, contain over 100 kcal/12-oz serving. Current recommendations to drink water for weight management do not define a context for drinking water goals in terms of other causal factors. They do not describe how drinking water fits into an overall food and drink pattern for weight management. It is unclear if plain water, *per se*, is necessary for weight change and/or if any particular dose of drinking water or condition is necessary to realize benefit.

Reinforcing uncertainty about the causal mechanism(s), drinking water interventions involving different populations and study conditions report conflicting effects on weight change. While some studies report that drinking water significantly increases weight loss (e.g., [24,25]), others report less weight gain (e.g., [21,26,27]), no effect (e.g., [28,29]) or less weight loss [30]. Variability across studies suggests the potential for disappointing intervention outcomes and the limited impact of global efforts to promote drinking water for weight management.

To date, recommendations to drink water for weight management have primarily been motivated by evidence that drinking water can decrease energy intake (EI). While drinking water can lower energy intake (e.g., [31]), it does not always have this effect. Under some conditions, drinking water has no effect (e.g., [32,33]) or even increases energy intake (e.g., [34]). Drinking water, furthermore, has heterogeneous effects on key intermediates besides energy intake, namely energy expenditure (EE) (e.g., [35,36,37,38]) and fat oxidation (FO) (e.g., [38,39,40]). If weight management recommendations and interventions do not specify conditions where drinking water lowers energy intake, increases energy expenditure and/or increases fat oxidation, null or negative effects of drinking water on weight change would not be surprising.

Reviews of beverage effects, to date, offer limited insight regarding conditions that might optimize the effects of drinking water for weight management, as they are focused on summarizing the magnitude of effect across studies [19,41,42,43]. Available reviews alert readers to significant between-study variation in effect size and outlier effects [19,41,42,43], but do not systematically evaluate factors associated with this heterogeneity. They suggest poor study quality [19], intervention setting or mode [44], young participant age [41] and the timing of drinking water (delay times of 30–80 min between drinking water and meals) [41] as potential reasons for null effects of drinking water and different hypotheses, research questions or parameters as reasons for inconsistent findings, without considering the determinants of weight change, such as background diet and/or activity conditions. Further research is needed to determine if, what and how background study conditions modify drinking water effects.

Short- and long-term effects of drinking water remain to be aligned and reconciled. Daniels and Popkin [41] point out inconsistent effects, such as significant weight change effects of drinking water for school-age children, despite short-term null effects of drinking water on energy intake for young children. Although reviews juxtapose short-term effects of drinking water on energy intake with longer-term effects of drinking water on weight change [19,41,45], they do not consider short-term effects of drinking water on energy expenditure or fat oxidation, which might also mediate weight change effects. Reviews not restricted to randomized trials are limited with respect to causal inference [19,41,45]. To elucidate and leverage any causal effect(s) of drinking water for weight management, further research is needed to align short-term effects of drinking water on energy intake, energy expenditure and fat oxidation with longer-term effects on weight.

As a preliminary step towards addressing gaps in knowledge about drinking water effects for weight management, this qualitative review aimed to identify diet and/or activity conditions associated with different short- and long-term effects of drinking water in the literature. Information about conditions associated with different drinking water effects may help to generate hypotheses about the underlying causal mechanism(s), inform the design of future meta-analyses, and ultimately optimize drinking water interventions for weight management.

## 2. Methods

### 2.1. Protocol Overview

One reviewer identified randomized, crossover experiments that report short-term (<24 h in duration) effects of drinking water on energy intake, energy expenditure or fat oxidation, and randomized controlled trials (RCT) that report longer-term (>4 weeks) effects of drinking water on weight change. The short-term period was chosen to focus on acute effects of drinking water, excluding delayed (e.g., 2–7 days) effects, which are potentially complex, due to metabolic, physiologic and/or psycho-social compensation. The longer-term period was chosen to exclude changes in body weight likely to be attributable only to shifts in body water. Drinking water effects on indices of body fat distribution (e.g., waist circumference, skinfold thickness), which may vary independent of energy intake, energy expenditure, fat oxidation, and total body weight were not included in this review. The same reviewer classified studies by outcome and extracted and analyzed the data.

### 2.2. Study Search

The PubMed database was searched for peer-reviewed article titles and abstracts, published in English up to December 2014, using the search terms “random”, “randomly” or “randomized”, “drinking water”, “water”, “fluid” or “placebo” and “energy intake”, “energy expenditure”, “thermogenesis”, “Resting energy expenditure (REE)”, “respiratory exchange ratio (RER)”, “respiratory quotient (RQ)”, “fuel partitioning”, “fat oxidation”, “weight change”, “weight loss”, “overweight” and “BMI”. The literature search was restricted to the PubMed database, because of the large number of studies for each of the four outcomes of interest. The goal of the review was to generate hypotheses about intervention conditions, not to estimate unbiased effects of drinking water. Additional studies were identified by searching the reference list of review articles [19,41,42,43,45,46,47], as well as the references cited by each identified article.

### 2.3. Study Inclusion and Exclusion Criteria

Only RCTs involving human participants and random assignment to a drinking water or control treatment were included in this review. RCTs were the focus of the review to restrict potential sources of error and facilitate inference about possible causal mechanisms, though RCTs were included in the review regardless of quality measures, such as double-blind design, complete protocol adherence, patient attrition, similarity of treatment and control groups at baseline and intention-to-treat analysis. RCTs were included in the review regardless of sample size, target population or unit of analysis (e.g., individual or group-level data). RCT differences in study design, protocol adherence, and/or sample characteristics may be informative with respect to conditions necessary for effect.

Drinking water exposures eligible for inclusion in this review were plain drinking water, tap or bottled water, carbonated or uncarbonated water, water served at any temperature, and placebo beverages, where plain water was matched for flavor, color, texture and/or electrolyte content with a caloric beverage using artificial, non-caloric additives. RCTs were eligible for inclusion if drinking water was tested alone, as a single exposure, or as part of a multi-component exposure. Effects of placebo beverages were tabulated separately from plain water effects and not excluded in the review, because they may shed light on whether or not oro-sensory mechanisms mediate the effects of plain drinking water. Although the effects of multicomponent interventions cannot be attributed solely to a change in drinking water, they may nevertheless shed light on multi-factorial conditions where drinking water interventions could impact weight change.

Studies were included in this review if they report at least one effect of drinking water on energy intake, energy expenditure, fat oxidation or body weight change. A statement in the text about the direction and significance of the effect, with or without an estimate of the magnitude of effect (*i.e.*, if the data are “not shown”), was sufficient for inclusion. For energy intake, studies were included in the review if they report an effect on food energy, beverage energy, meal energy or daily total energy intake in absolute or relative (expressed relative to baseline or body weight) units. For energy expenditure, studies were included if they report effects of drinking water on oxygen consumption, resting energy expenditure, postprandial thermogenesis, physical activity energy expenditure or daily total energy expenditure. For fat oxidation, studies were included if they report the rate of fat oxidation or a measure of relative fuel partitioning, respiratory quotient (RQ) or respiratory exchange ratio (RER). With respect to body weight change, studies were included if they report change in absolute weight, relative weight (BMI, BMI percentile, BMI z-score) or prevalence of overweight and/or obesity. Outcome variables could be specified at the individual, group or population level. Studies reporting weight change over periods longer than one month were included, as these more likely reflect shifts in body fat as opposed to body water.

Although some authors hypothesize that intake of sugar-sweetened beverages instead of healthy alternatives, such as water, may increase the risk of obesity by increasing hepatic *de novo* lipogenesis [48,49,50,51], the effects of drinking water on fat synthesis were not included in this review, because the magnitude of fat synthesized from excess carbohydrate intake is negligible [52,53,54,55]. Absolute hepatic *de novo* lipogenesis accounts for less than 5 g of fatty acids synthesized per day, even when the diet contains +50% surplus carbohydrate [56]. Excess carbohydrate intake results in an increase in body fat stores, not by conversion of carbohydrate to fat, but rather by suppressing the oxidation of dietary fat [57]. Mechanisms other than the provision of substrates for *de novo* lipogenesis explain the lipogenic effects of sugar-sweetened beverages [58].

### 2.4. Data Extraction and Classification

Data were systematically extracted from each study using a standardized table that captured information about the type of drinking water exposure and study conditions. Each line in each table represented one exposure and condition-specific effect. Studies that tested multiple drinking water exposures involved multiple population groups or conditions and/or observed effects of drinking water on multiple outcomes were represented by multiple rows of data.

Each drinking water exposure was classified as a relative and/or absolute exposure. Relative exposures compare drinking water with the same specified volume of another beverage. Absolute exposures compare a larger volume of drinking water with a smaller volume of drinking water. Both exposures are potentially feasible or relevant for public health intervention, because other beverage intake accounts for over 40% of total water intake in free-living adults and children [59,60], and healthy individuals can experience limited access to water [61], involuntary dehydration [62] and/or thirst deficit [63] under daily life conditions.

Age, sex, weight, health status, diet and exercise conditions were considered in this review, because these variables determine the water requirements and water intake of free-living individuals [20,64], modify fat oxidation [65,66] and determine body weight. Although smoking also determines water requirements and water intake, smoking was not systematically extracted in this review, because RCTs typically recruit non-smokers and/or prohibit smoking during the study period. Weight status was indexed as normal weight (BMI: 18.5–24.9) or overweight or obese (BMI ≥ 25.0). To allow diet conditions in the short-term experiments to be compared with those specified in longer-term interventions, diet conditions were classified as *ad libitum* or restricted, fed or fasting. Physical activity conditions were classified as sitting at rest, low- or moderate-intensity exercise or high-intensity exercise.

### 2.5. Data Analysis

For RCTs that report multiple effects of drinking water on different outcomes and/or for different study groups, each effect was considered separately. Each effect was grouped according to the type of RCT and type of outcome (energy intake, energy expenditure, fat oxidation or weight loss) and sub-grouped by the type of drinking water effect (e.g., negative, null or positive), drinking water exposure (absolute or relative), diet and physical activity conditions. Single-component weight change interventions were distinguished from multicomponent interventions. The effects of plain drinking water were distinguished from the effects of placebo beverages (water matched for color, flavor, electrolytes and/or sweetener). To the extent possible, effects were classified based on the observed diet and activity. If, for example, an intervention aimed to alter dietary intake, but reported no significant change in dietary intake, then the effect was grouped with effects from studies that had usual or *ad libitum* background diet.

To avoid over-representing conditions from RCTs reporting multiple effects relative to conditions from RCTs reporting only one effect, no weight was given to the number of effects reported for a given condition. The drinking water exposure(s), study population(s) and background diet and activity condition(s) associated with each type of outcome were described in qualitative terms. Exposures and conditions associated with lower total energy intake, greater energy expenditure, greater fat oxidation, less weight gain and greater weight loss were identified.

The working hypotheses for this analysis were that conditions associated with null effects in short-term studies would be associated with null effects in longer term studies, and conversely, conditions associated with lower total energy intake, greater energy expenditure and/or fat oxidation would be associated with less weight gain or weight loss in longer term studies.

## 3. Results

### 3.1. Drinking Water Effects on Energy Intake

Table 2 summarizes the exposures and conditions associated with 185 effects of plain drinking water on energy intake in RCTs (see Appendix 1 for further detail). Drinking water reportedly lowers energy intake in over 60 instances, has no effect in over 90 instances and increases energy intake in 30 instances.

The studies in Table 2 typically employ a preload test meal design, where each participant serves as his/her own control and repeatedly returns to the laboratory setting on different days to consume a different preload beverage (or no beverage) before and/or with the same test meal. In general, these studies standardize the dietary intake, physical activity, smoking, alcohol, caffeine and medication use for 24 h before each test day. With a few exceptions (e.g., studies that include restrained eaters [32,67]), participants have unrestricted diets, no binge or disordered eating, no allergies, no depression and no disease or medications that affect appetite. The studies range with respect to background levels of usual physical activity. Some studies explicitly exclude individuals participating in more than 150 min/week of vigorous physical activity [68,69]. Others select for physically-active participants (>3 h/week exercise) [70]. The participants sit at rest in the majority of studies. Participants consume the test meal in the laboratory, but are allowed to behave as normal between test meal periods.

### 3.2. Drinking Water Lowers Energy Intake

Drinking water lowers energy intake when drinking water is compared with the same volume of a caloric beverage. The effect is observed when drinking water is compared with various carbohydrate solutions, 100% juice, sweetened milk, unsweetened milk, soda and alcoholic beverages. The studies test solutions mixed in the laboratory, as well as commercially available beverages. The studies test drink volumes ranging from 0.2 to 0.5 L. They test time intervals ranging from no delay between beverage and test meal to a 2-h delay between beverage preload and test meal.

RCTs reporting this effect consistently involve a condition of *ad libitum* food intake in disinhibited or unrestrained eaters. When non-dieting, unrestrained eaters are given a caloric test beverage before and/or during a meal of *ad libitum* food, they do not completely compensate for the beverage calories by eating less food than they do when the same meal is paired with drinking water [41,46]. In some studies (e.g., [31]), they, in fact, eat the same amount of food, regardless of the type of beverage paired with the meal, such that the beverage calories add on top of food energy, resulting in a net total caloric excess for the meal that is approximately equivalent to the preload beverage energy content.

Young children compensate for preload calories by decreasing the amount of food consumed [71,72]. The compensation may be incomplete, however, such that total meal energy intake remains significantly elevated relative to the same meal paired with drinking water [71,72]. Pre-school children, ages 20–56 months, consume large quantities of chocolate milk when it is offered, without decreasing their intake of other food items, and significantly increase total energy intake [73]. Children ages 4–6 years consume 17%–26% more energy at lunch when plain milk is offered [72]. Summarizing across studies, when sugar-sweetened drinks are consumed within 0–30 min of a meal or juice or milk are consumed within 2 h of a meal, the total meal energy intake is on average 10%–15% higher than the same meal paired with drinking water [41].

Lower energy intake after drinking water instead of caloric beverages does not appear to be explained by oro-sensory effects. The effect is observed in RCTs that compare caloric beverages with plain water, matched for color, flavor and/or electrolyte content (highlighted in grey in the Appendix).

Table 2 includes five reports of drinking more *vs.* less plain water lowering energy intake, before or during an *ad libitum* test meal. Three of these effects [25,74,75] are observed with 0.5 L *vs.* no water in overweight or obese, older adults 30 min before a test meal, following 12 or more hours of food and water restriction. The effects are reported for participants who were mildly dehydrated, with a urine-specific gravity over 1.010, and thirsty, before the preload. Thirst ratings decrease significantly after the 0.5 L water [25]. The other two effects [67,68] are observed in normal weight, younger adults, consuming 0.3 L more drinking water between mouthfuls of food, after a standardized breakfast and 4 h of water restriction.

In sum, the diet and activity conditions associated with lower energy intake after drinking water are *ad libitum* food and sitting at rest.

### 3.3. Drinking Water Has No Effect on Energy Intake

Forty-two null effects of drinking water on energy intake were identified in association with drinking water instead of a non-calorically sweetened or “diet” drink (see Table 2). Energy intake following drinking water did not differ from energy intake after an equivalent volume of sucralose, aspartame, saccharin or xylitol for adult men and women, regardless of weight status, given *ad libitum* diet and resting conditions.

Birch *et al.* [76] report that drinking water and sucrose preloads result in the same energy intake (beverage preload plus test meal foods) in young children ages 2–5 years. They observe that the children compensate for the preload calories by decreasing intake of test foods that are not preferred foods [76]. The ability to compensate may reflect the children’s opportunity to control their own food intake [77] and/or food preferences [78]. Children reportedly continue to consume foods they prefer, while eliminating non-preferred foods after higher energy preloads [78].

Lavin *et al.* [79] report no effect of drinking water instead of a sucrose-sweetened beverage on energy intake in restrained eaters. Individuals who restrict their food intake consciously decrease the amount of food consumed to compensate for beverage calories.

RCTs report null effects of drinking water instead of acid whey or fructose solutions of preload plus meal energy intake (e.g., [34]).

Under conditions of *ad libitum* food, many RCTs report no effect of drinking water instead of caloric beverages on food intake, as distinct from total meal, food plus beverage, calories (24 null effects in Table 2).

**Table 2:** RCT conditions associated with negative, null and positive effects of plain drinking water on energy intake.

Effect of Drinking Water	Number of Effects	Type of Drinking Water Exposure	Reference Condition	Participant Age, Sex	Participant Weight Status	Diet Condition	Activity Condition
Lower energy intake	56	Relative	Same volume of: glucose; fructose; sucrose; mixed carbohydrate; whey; infant formula; soy milk; milk; juice; cola; soda wine; beer	4–65 years M, F	N, O	*Ad libitum* food	Rest
	5	Absolute	0.5 L *vs.* 0 L 0.4 L *vs.* 0.3 L	55–80 years M, F	O, N	*Ad libitum* food, 12 h water restricted, between mouthfuls of food	Rest
No effect	42	Relative	Same volume of: aspartame; acesulfame-K; sucralose; saccharin; diet cola	2–56 years M, F	N, O	*Ad libitum* food	Rest or usual
	24	Relative	Same volume of: glucose; high fructose corn syrup; sucrose; infant formula; soy milk; milk; juice; soda; wine; beer	18–50 years, M, F	N, O	*Ad libitum* food	Rest or usual
	9	Relative	Same volume of: fructose; whey protein	19–50 years M, F	N, O	*Ad libitum* food	Rest
	16	Absolute	0.3–0.6 L *vs.* 0–0.3 L, 100% *vs.* 40% of usual	19–95 years M, F	N, O	*Ad libitum* food, not water restricted	Rest
	1	Absolute and Relative	560 mL aspartame soft drink *vs.* 280 mL water	19–25 years M	N, O	*Ad libitum* food	Rest
Higher energy intake	28	Relative	Same volume of: whey; aspartame; fructose; xylitol	2–65 years M, F	N, O	*Ad libitum* food	Rest or usual
	2	Absolute	Euhydrated *vs.* 3% BWT loss; 100% *vs.* 43% of usual	20–25 years M	N	*Ad libitum* food, dehydrated	After exercise

RCT, randomized controlled trial; N, normal weight; O, overweight or obese; usual, level in daily life; BWT loss, acute weight loss due to exercise or sauna.

Some RCTs that report null effects of drinking water on energy intake test the effect of a small (0.3 L) difference in absolute volume in normal weight or non-obese participants. No significant difference in energy intake is observed when drinking water is served 90 min [32], 60 min [80], 30 min [75,80,81] or 5 min before the test meal [82] or when the water is served with the test meal foods [31,33,80], without specific instructions about when or how to drink the water and without a meal time constraint (20 min for the meal or no time limit specified). Compared to no drink, 0.5 L drinking water has no effect on energy intake in overweight, obese and older after 12 weeks of a hypo-caloric diet intervention, contrary to the significant effect observed before the diet intervention [25]. There is no absolute effect of drinking water on food intake when overweight and obese individuals consume more *vs.* less water between mouthfuls of food, contrary to what is observed in normal weight adults [68].

### 3.4. Drinking Water Increases Energy Intake

RCTs that report higher total energy intake after drinking water compared drinking water with laboratory solutions of whey protein [34,83], fructose alone [84,85] and fructose mixed with glucose (F80:G20) after an 80-min delay [86]. In these studies, the whey protein, fructose or fructose-glucose beverages cause participants to have significantly greater blood insulin concentrations with minimal or no increase in blood glucose immediately before they consume the test meal [83,85,86]. Insulin response and glucose uptake are thought to play a role in the suppression of food intake following fructose or whey protein [87,88], but the mechanism remains to be elucidated [89].

Pure fructose solutions may suppress food intake to a greater extent than drinking water for reasons besides insulin response. The slow absorption of fructose may prolong its contact with gastrointestinal receptors that signal satiety [90]. When ingested alone, incomplete absorption of fructose produces a hyperosmolar environment in the large intestine that causes discomfort, diarrhea and nausea [91].

One RCT in young, normal weight humans, given *ad libitum* food after exercise, reports that drinking water results in higher energy intake when 100% of usual water intake is compared with 43% of usual intake over 48 h [92]. These data in humans [92] are consistent with findings across species that food intake is positively associated with hydration state [93,94,95] (note: results from studies in animals are not included in Table 2, which summarizes effects in humans only). In animals, drinking water more *vs.* less increases, rather than decreases, food intake, if the drinking water results in a hydrated *vs.* dehydrated state. Extended periods of cellular dehydration result in anorexia, due to physiological adaptations that limit the intake of osmolytes from food. “Dehydration-anorexia” is mediated by neural networks that control meal termination [96,97].

### 3.5. Drinking Water Effects on Energy Expenditure

Table 3 summarizes RCT conditions associated with 68 short-term effects of plain drinking water on oxygen consumption or energy expenditure. Drinking water has negative, null and positive effects on oxygen consumption or energy expenditure.

The RCTs in Table 3 are crossover experiments that typically involve multiple test days for each participant, separated by 4–7 days. The order of the test days is randomly assigned. The tests are performed after 24 h of standardized dietary intake, physical activity, smoking and medication use, at the same time of day, with standardized temperature and humidity. Many, but not all, studies familiarize the participants with equipment and procedures before the test. Food intake before each test day is standardized by food records in the majority of studies, though some protocols provide food in the research center [38,98,99]. Participants refrain from alcohol and strenuous exercise before each test day. Some studies require participants to avoid corn and sugar intake before each test day to allow the measurement of exogenous carbohydrate oxidation using a glucose stable isotope (13C-labelled glucose). Some study protocols involve muscle biopsy [100,101,102,103] or serial blood collection via catheter or thumb pricks [104,105]. Drinking water is provided as a bolus before the test or in bouts every 10–20 min before and during the test. Tests are performed under ambient conditions or in heat (35C 40%–50% humidity) [39,106,107,108], with participants sitting at rest or performing exercise. Exercise protocols vary in intensity and involve cycling, treadmill walking or running and arm crank tests.

**Table 3:** RCT conditions associated with negative, null and positive effects of plain drinking water on oxygen consumption or energy expenditure.

Effect of Drinking Water	Number of Effects	Type of Drinking Water Exposure	Reference Condition	Participant Age, Sex	Participant Weight Status	Diet Condition	Activity Condition
Lower VO_2_ or EE	9	Relative	Same volume of: sucrose; glucose; fructose; whey; milk; juice; soft drink	20–40 years, M, F	N	Fasting	Rest
	2	Relative	Same volume of: caffeine; oolong tea	25–60 years, M	N	Fed	LM
	2	Absolute	2 L *vs.* no fluid; drinking to thirst *vs.* no fluid and IV NaCl	20–30 years, M	N	Fasting	Rest
No effect	33	Relative	Same volume of: glucose; fructose; trehalose; isomaltulose; amylopectin; maltodextrin; caffeine, sports drink; milk; oats	20–40 years, M, F	N	Fasting	LM, H
	2	Relative	Same volume of: orange juice	10–40 years, M, F	N	Fed	Rest
	9	Relative	Same volume of: glucose; fructose; caffeine; sports drink	20–40 years, M, F	N	Fed	LM, H
	2	Absolute	2 L *vs.* *ad libitum*; 2.4 L *vs.* no fluid	20–30 years, M	N	Fasting	Rest, LM
	2	Absolute	1.2–2.4 L *vs.* no fluid	20–30 years, M, F	N	Fed	LM
Higher VO_2_ or EE	5	Relative	Same volume of: glucose; fructose	20–40 years, M, F	N, O	Fed	LM
	1	Relative	Same volume of: saline	20–42 years, M, F	O	Fasting	Rest
	1	Absolute	0.5 L *vs.* 50 mL	20–42 years, M, F	O	Fasting	Rest

RCT, randomized controlled trial; VO_2_, oxygen consumption; EE, energy expenditure; N, normal weight; O, overweight or obese; rest, lying supine or sitting at rest; LM, low- or moderate-intensity exercise; H, high intensity exercise.

### 3.6. Drinking Water Lowers Energy Expenditure

Drinking water lowers oxygen consumption or energy expenditure when drinking water is compared with the same volume of a caloric beverage in participants who are fasting and sitting at rest. The greater energy expenditure associated with caloric beverages is due to the thermic effect of the beverage macronutrients, which is proportional to the beverage macronutrient absorption and availability. Although the rate of energy expenditure is significantly lower after drinking water than it is after caloric beverage intake in these studies, it is important to note that the net energy balance is not lower after drinking water. The increase in energy expenditure associated with caloric beverages is smaller than the energy content of the beverages [35,40]. Li *et al.* [35], for example, report a thermic effect of only 0.48 kJ/min following 280 mL of Sprite containing 538 kJ.

Drinking water reportedly also lowers energy expenditure relative to the same volume of a non-caloric, caffeinated beverage (oolong tea) under non-fasting, low- to moderate-intensity exercise conditions [38,109].

### 3.7. Drinking Water Has No Effect on Energy Expenditure

Drinking water has no effect on energy expenditure in studies that test relative or absolute effects of drinking water in normal weight individuals, under fed and/or exercising conditions. The lack of difference in energy expenditure or change in energy expenditure between treatments may be explained by a fixed thermic response to food intake and/or fixed energy expenditure demands for exercise. Null effects on how much energy is expended do not rule out effects on fuel partitioning (e.g., [36]) (see below).

### 3.8. Drinking Water Results in Higher Energy Expenditure

The literature search identified one RCT that reports that 0.5 L plain drinking water results in higher energy expenditure than the same volume (0.5 L) of saline or 50 mL plain water in overweight or obese participants, who are fasting and sitting at rest [37]. The authors propose that the effect of drinking water *vs.* saline is due to its low osmolality. Drinking water has a concentration far below that of blood (osmolality < 20 mmol/kg *vs.* 280 mmol/kg), which allows it to move into cells, following local osmotic gradients, and acutely swell cells, which in turn activates organ function and/or osmosensitive neural pathways.

Boschmann *et al.* [37] further observe that the “RQ did not change significantly…Obese subjects may be less able to switch between carbohydrate and lipid oxidation”. They speculate that metabolic inflexibility or altered regulation in obesity might account for between-study differences in effect. Consistent with this hypothesis, in non-randomized, pre-post studies of water ingestion in obese participants, drinking water is associated with greater energy expenditure, with no change in RQ [110].

A randomized crossover study involving obese boys, ages 9–12 years, reports significantly higher energy expenditure together with significantly lower RQ during exercise, 3 h after eating, when water mixed with Splenda and 9 mM NaCl is consumed instead of a 6% glucose solution [111].

Blacker *et al.* [112] compare 250 mL flavored water (placebo) with 250 mL 6.4% carbohydrate beverage in healthy men walking on a treadmill, carrying a 25-kg backpack. The placebo results in greater oxygen consumption and lower RER than the carbohydrate beverage.

### 3.9. Drinking Water Effects on Fat Oxidation

Table 4 summarizes the exposures and conditions associated with 74 effects of plain drinking water on fat oxidation in human study participants. The effects are stratified by negative, null and positive outcome. The crossover design and typical protocol of these experiments is described above.

**Table 4:** RCT conditions associated with negative, null and positive effects of plain drinking water on fat oxidation.

Effect of Drinking Water	Number of Effects	Type of Drinking Water Exposure	Reference Condition	Participant Age, Sex	Participant Weight Status	Diet Condition	Activity Condition
Lower fat oxidation	2	Relative	Same volume of: oolong tea; caffeine	25–60 years M	N	Fed	LM in 24-h room calorimeter
No effect	3	Relative	Same volume of: Powerade; milk; milk and glucose	20–30 years M	N	Fasting	H
	3	Relative	Same volume of: maltodextrin; sucrose; caffeine	20–30 years M	N	Fed	LM
Higher fat oxidation	33	Relative	Same volume of: glucose; sucrose; maltodextrin; mixed CHO; sports drink; oats	20–40 years M, F	N	Fasting	LM, H
	19	Relative	Same volume of: glucose; fructose; maltodextrin; sports drink; caffeine	20–40 years M, F	N	Fed	LM
	6	Relative	Same volume of: glucose; fructose; sucrose; milk; orange juice	21–33 years M, F	N	Fasting	Rest
	2	Relative	Orange juice	11–38 years M, F	N	Fed	Rest
	1	Absolute	No fluid	20–25 years F	N	Fed	LM
	3	Absolute	No fluid	20–30 years M	N	Fasting	LM, H
	3	Absolute	No fluid and IV NaCl; 50 mL; *ad libitum* fluid	20–42 years M, F	N, O	Fasting	Rest

RCT, randomized controlled trial; N, normal weight; O, overweight or obese; rest, lying supine or sitting at rest; LM, low- or moderate-intensity exercise; H, high intensity exercise.

### 3.10. Drinking Water Results in Lower Fat Oxidation

Drinking water results in lower fat oxidation over 24 h relative (including meal periods) to the same volume of oolong tea, in normal weight adults, studied in a room calorimeter [38].

### 3.11. Drinking Water Has No Effect on Fat Oxidation

Drinking water has no effect on fat oxidation in RCTs that compare drinking water with the same volume of carbohydrate solutions, sports drinks and milk in young, normal weight, trained athletes, during high-intensity exercise. High-intensity exercise is dominated by carbohydrate oxidation, regardless of dietary manipulation and substrate availability [113], because carbohydrate and creatine phosphate are the only fuel sources capable of rapid ATP resynthesis when oxygen delivery to the muscles is compromised.

### 3.12. Drinking Water Results in Greater Fat Oxidation

Drinking water results in greater fat oxidation when drinking water is compared with the same volume of a caloric reference beverage: a glucose, fructose, sucrose, whey or mixed carbohydrate solution or commercial sports drink. This relative effect is observed in studies involving men, women and children, in athletes and untrained individuals, when participants are sitting at rest or doing 60–120 min of exercise <70% VO_2_ max. This relative effect is observed with different beverage volumes, consumed before and/or during the exercise, in the fed or fasted state.

Greater fat oxidation after drinking water instead of caloric beverages occurs in association with lower blood glucose, lower insulin concentrations, lower heart rate and/or higher free fatty acid concentrations. The effect is attributable to the fact that drinking water has a glycemic index of zero and an insulin index of zero, so does not inhibit the rate-limiting biochemical steps for fat breakdown to free fatty acids, transport of free fatty acids into the mitochondria and fat oxidation, like other beverages that contain carbohydrate and/or trigger insulin. Even small amounts of fructose are enough to significantly suppress fat oxidation [114]. Compared to plain water, milk significantly increases postprandial insulinemia; “Even an ordinary amount of milk (200 mL) increase[s] the insulin AUC to the same level as seen with white bread [115]”.

Drinking water results in greater fat oxidation in normal weight individuals who are sitting at rest after a meal, despite positive net energy balance for hours after the meal [40]. A meal paired with 0.5 L drinking water, for example, contains 365 kcal, 12 g fat and 55 g carbohydrate. The same foods paired with an equal volume of orange juice contains 575 kcal, 12 g fat and 106 g carbohydrate. Energy consumed at both breakfasts exceeds energy expended in the subsequent 150 min on both test days. Fat oxidation is, nevertheless, significantly less suppressed after the meal with drinking water, resulting in a significantly more negative fat balance 150 min after the meal. The estimated net fat balance for adolescents is −7 g (12 g consumed, 19 g oxidized) after the meal with drinking water compared to −2 g after the meal with orange juice. For the adults, the estimated net fat balance is negative after the meal with drinking water, but positive after the meal with orange juice.

Absolute effects of drinking water on fat oxidation are reported by studies that compare 0.5–3.5 L drinking water *vs.* no fluid intake in young, normal weight, trained athletes. The participants are fed or fasting, sitting at rest or doing moderate-intensity exercise.

The greater fat oxidation after drinking water in these studies is associated with lower serum osmolality, lower blood glucose and insulin concentrations, lower proteolysis and less muscle glycogen use [36,116,117,118,119]. The effect, which is independent of beverage macronutrient content, may be attributed to cell swelling. Drinking water is different from many other beverages in this respect. The osmolality of Pepsi (568 mmol/kg), Coca-Cola (650 mmol/kg) and fruit juice (690 mmol/kg), for example, is about double the normal osmolality of blood.

Cell swelling favors fat oxidation by not stimulating glycogen breakdown, proteolysis and gluconeogenesis [120,121,122]. At the same time, cell swelling also improves insulin sensitivity and glucose clearance. Cell swelling promotes insulin sensitivity by mediating the effects of insulin. Insulin swells hepatocytes by activating Na^+^/H^+^ exchange and Na^+^K^+^2Cl^−^ cotransport [123,124]. Cell shrinkage impairs insulin signaling and “may be a major cause of insulin resistance which develops in systemic hyperosmolality” [123]. Drinking water does not deliver exogenous carbohydrate or amino acids, does not trigger the production of endogenous carbohydrate or free amino acids and also accelerates blood glucose clearance.

### 3.13. Drinking Water Effects on Weight Change

Table 5 and Table 6 describe the weight change effects of plain drinking water from single-component and multi-component RCTs conducted in the USA, U.K., Germany, the Netherlands, Belgium, Denmark, Portugal, Brazil, Mexico, Australia and Iran. Drinking water is reported to result in less weight loss, no difference, less weight gain and more weight loss.

With few exceptions [125,126], the RCTs in Table 5 and Table 6 were unblinded and heterogeneous in terms of the unit of analysis, sample size, exposure and outcome specification, measures, protocol adherence, loss to follow-up and analysis. Communities, schools, mother-child pairs or individual children are randomly assigned to intervention or control treatment groups. Weight change effects are defined in terms of change in weight (kg or lbs), percent body fat, BMI, BMI percentile, BMI z-score, individual weight status, group mean status, risk or prevalence of overweight and overweight incidence. Treatment and control groups are compared using intention-to-treat, sub-group and/or completer analyses.

### 3.14. Drinking Water Results in Less Weight Loss

Drinking water results in less weight loss than the same volume (at least 24 fl oz) of non-nutritive sweetened beverages (NNS) in one single-component RCT [30]. This study reports the effects of drinking water instead of NNS beverages on absolute (kg) and relative (% of participants losing >5% of body weight) weight loss over three months in overweight or obese adults who initially consumed NNS beverages daily. The protocol restricts energy intake by providing each participant comprehensive cognitive behavioral weight loss counseling, including a set energy intake target for weight loss. The authors note that “assignment to the NNS treatment did not require as great a behavior change as the Water group who had to abstain from NNS beverages” for the duration of the 12-week trial [30].

### 3.15. Drinking Water Has No Effect on Weight Change

Drinking water instead of sugar-sweetened beverages (SSB) has no significant effect on weight change in normal weight, overweight or obese children, with *ad libitum* diet and usual exercise. Sichieri *et al.* [127] test a school-based program of simple messages to encourage the intake of drinking water instead of sugar-sweetened carbonated beverages (SSBs). Ebbeling *et al.* [128] test instructions to replace SSBs with non-caloric beverages, with home delivery of drinking water and non-caloric beverages. Sichieri *et al.* [127] report that SSB intake in the intervention schools only decreased by a small amount (67 mL/day), and the change was offset by an increase in juice intake: “The small change was associated with compensation… due to an increase in juices”. Ebbeling *et al.* [128] observe that “BMI changes did not differ… among subjects with lower baseline body weight, (who had) lower baseline energy intake from SSBs”.

Drinking water instead of NNS does not result in significantly different weight loss in overweight or obese adults with *ad libitum* diet and usual physical activity [22,69]. Tate *et al.* [22] observe that the “reduction of caloric beverages was approximately (the same in both groups).There were no between group differences in energy intake from food.”

**Table 5:** Single-component RCT conditions associated with negative, null and positive effects of plain drinking water on weight change.

Effect of Drinking Water	Number of Effects	Duration	Type of Drinking Water Exposure	Reference Condition	Participant Age, Sex	Participant Weight Status	Diet Condition	Activity Condition
Less weight loss	2	12 weeks	Relative	Non-nutritively-sweetened beverages	21–65 years M, F	O	Restricted	Increase
No effect	24	25 weeks 1 year	Relative	Sugar-sweetened beverages; Milk; Non-caloric drinks	7–50 years M, F	N, O	*Ad libitum*	Usual
	7	6 months	Relative	All caloric beverages; Sugar-sweetened beverages; Diet cola	18–65 years, M, F	O	Restricted	Usual
	1	1 school year	Absolute	Groups differed by: 1 glass/day water	7–9 years M, F	N, O	*Ad libitum*	Usual
	2	8 weeks	Absolute	Groups differed by: 1.5 L/day, 297 mmol/kg Urine osmolality	9–12 years, 55–75 years M, F	O	Restricted	Usual
Less weight gain	8	12 weeks 1 year	Relative	Skim milk; Sugar-sweetened beverages; Sugar-sweetened beverages and Juice	7–15 years M, F	O	*Ad libitum*	Usual
	1	1 year	Relative	“Fizzy drinks”	7–11 years M, F	N,O	*Ad libitum*	Usual
	1	1 school year	Absolute	Groups differed by: 1 glass/day	7–9 years M, F	N, O	*Ad libitum*	Usual
More weight loss	1	25 weeks	Relative	Sugar-sweetened beverages	13–18 years M, F	O	*Ad libitum*	Usual
	3	8 weeks 12 weeks, 12 months	Absolute	Groups differed by: 1 L/day drinking water; 706 mmol/kg Urine osmolality	9–12 years, 55–75 years M, F	O	Restricted	Usual, increase

RCT, randomized controlled trial; N, normal weight; O, overweight or obese.

**Table 6:** Multiple component RCT conditions associated with negative, null and positive effects of plain drinking water on weight change.

Effect of Drinking Water	Number of Effects	Duration	Type of Drinking Water Exposure	Reference Condition	Participant Age, Sex	Participant Weight Status	Diet Condition	Activity Condition
No effect	19	3–24 months	Relative	Sugar-sweetened beverages	2–14 years M, F	N, O	*Ad libitum*, not-restricted	Usual
	8	6–12 months	Relative	Sugar-sweetened beverages and juice; >1% milk and sugar-sweetened beverages and juice	3–6 years M, F	N, O	Increased F and V, less fast food	Usual, less screen time
	22	1–2 school years	Relative and absolute	Sugar-sweetened beverages and usual water	11–15 years M, F	N, O	Increased F and V	Increased
Less weight gain	4	6-12 months	Relative	Sugar-sweetened beverages	4–13 years M, F	N, O	Ad-libitum	Usual
	2	3 school years	Relative	>1% milk; sugar-sweetened beverages and juice	10–14 years M, F	N, O	Increased F and V	Increased
	4	6 months	Absolute	No effort to drink water during break	10–13 years M, F	N, O	Increased F and V	Usual
	1	18 months	Relative and absolute	Sugar-sweetened beverages and milk and usual water	Adult M, F	O	*Ad libitum*	Usual
	3	2 school years	Relative and absolute	Sugar-sweetened beverages and usual water; sugar-sweetened beverages and >1% milk	11–15 years F	N, O	Increased F and V	Increased
More weight loss	1	6 months	Relative	Sugar-sweetened beverages	6–12 years M, F	O	*Ad libitum*	Usual
	1	12 months	Relative	Sugar-sweetened beverages	9–24 months M,F	N, O	Increased F and V	Increased

Drinking water instead of caloric beverages has no significant effect on weight change in overweight or obese participants who restrict food intake [28,69]. Hernandez-Cordero *et al.* [28] provide monthly face-to-face meetings with a dietician for each woman to identify a healthy diet goal. The authors note that “the (control) group tended to have a greater decrease in energy intake from solid foods. Thus… the average total energy did not differ (between groups)”. Maersk *et al.* [69] compare drinking 1 L/day water instead of the equivalent volume of cola or 2% milk in 47 overweight or obese adults. They report no difference in total energy intake between the treatment groups and note that “the consumption of regular cola and milk… was generally compensated for by reducing energy intake from other sources… The study was unblinded, which may have affected the subjects’ behavior to counteract some of the expected effects of the beverages, such as weight gain in the energy-containing beverage groups.”

Small absolute increases in drinking water have a null effect on weight change [27,129]. Muckelbauer *et al.* [27] increase drinking water by one glass/d in normal weight, overweight and obese children, with *ad libitum* diet and usual exercise conditions. The participants maintain their background caloric beverage intake, with “no intervention effect on juice and soft drink consumption”. Stookey *et al.* [129] increase drinking water by one 500-mL bottle/day, enough to dilute urine by only 148 mmol/kg, not enough to dilute the mean urine osmolality below 725 mmol/kg over the eight-week study period. In this RCT, “participants reported lack of thirst as barriers to drinking more water.” Participants in this study were overweight or obese children, with a restricted diet (limited caloric beverages and high glycemic foods) and usual physical activity levels [129].

Multi-component interventions that report null effects on weight target reduction of SSB intake only fail to increase intake of drinking water and/or do not decrease caloric beverage intake report, despite protocol intent to alter beverage intake (see the Appendix).

### 3.16. Drinking Water Results in Less Weight Gain

Drinking water results in less weight gain in RCTs that involve *ad libitum* diet and usual physical activity. The studies vary with respect to drinking water exposure and participant population. Less weight gain is associated with drinking water instead of SSBs and juice [21,127] and drinking water instead of skim milk [130] in overweight or obese participants. In school-based RCTs, which involve all students, regardless of weight status, smaller increases in the prevalence of overweight is associated with drinking relatively more water and less “fizzy” drinks [26] and one glass/d more drinking water [27]. Absolute increases in intake of drinking water intake, without change in absolute caloric beverage intake, is achieved by teachers promoting the filling of water bottles in the morning and intake of drinking water during class [27]. Muckelbauer *et al.* [27] note that “children with a body weight close to the cutoff point for overweight received the greatest benefit”.

Ebbeling *et al.* [21] suggest that decreases in sugar intake from beverages mediate the effect of drinking water instead of SSBs: “When sugar intake was added to the model, the intervention effect on BMI was strongly attenuated and no longer significant”. Arnberg *et al.* [130] and their study participants believed that “a high intake of milk protein would reduce body weight… (and) were surprised to see an increase in the (BMI-for-age Z-scores) BAZ following skim milk”. Given their belief and expectations, conscious compensation or food intake regulation is an unlikely explanation for less weight gain after drinking water instead of skim milk, though the study was unblinded.

In multi-component RCTs involving children, under conditions of *ad libitum* diet and usual activity, drinking water instead of SSB, juice and milk and absolute increases of drinking water during break time result in less weight gain.

### 3.17. Drinking Water Results in More Weight Loss

Drinking water instead of SSBs results in more weight loss in RCTs that involve overweight or obese participants with *ad libitum*, usual food intake and usual physical activity levels. The effect is not attributed to calories from SSB only; the authors comment that “the greater weight loss… was not simply because of a greater decrease in energy intake from SSBs. Perhaps some individuals are inherently more susceptible than others [128]”.

Large absolute increases in drinking water are associated with significantly greater weight loss in overweight participants who significantly dilute urine and restrict diet [25,129,131]. The effects are not solely attributed to decreases in total energy intake. Dennis *et al.* [25] observe that “beverage energy intake declined by 100 kcal, but did not differ between groups and is thus unlikely to explain our findings. We did not detect group differences in total energy intake”. Akers *et al.* [131] observe that self-monitoring of pre-meal water consumption may be an effective approach, beyond that achieved by daily monitoring of body weight, step count, and fruit and vegetable consumption”. Stookey *et al.* [129] observe that the study participants who drank enough water to dilute urine below 500 mmol/kg decreased their saliva insulin by 81% to below 15 pmol/L over the last three weeks. Saliva insulin was above this value for study participants who did not drink enough water to dilute urine, despite similar adherence to the study foods. The decrease in saliva insulin partially mediated the association between diluting urine osmolality below 500 mmol/kg and weight loss.

One multi-component RCT reporting that drinking water results in more weight loss included three times as many overweight children in the intervention group than the control group [132]. The study included toddlers, who normally have a decrease in BMI as they become more mobile. Parents were instructed to “put water on the table during every meal”. Water intake increased significantly more in the intervention group (by +10% *vs.* +6% of beverage intake), while treatment groups did not differ with respect to the change in the intake of soft drinks, sweet milk, unsweetened milk, fruit, vegetables, sweets, snacks, physical activity or screen time. Another multi-component RCT reporting more weight loss attributes the greater weight loss to changes in overweight children close to the cutoff for normal weight [133].

## 4. Discussion

The goal of this literature review was to explore for RCT conditions associated with different effects of drinking water on energy intake, energy expenditure, fat oxidation and body weight. Awareness about sources of heterogeneity in drinking water effects may generate hypotheses about ways to optimize drinking water interventions for weight management, to maximize the impact of global investments in drinking water initiatives.

The literature review identified over 440 effects of plain drinking water on energy intake, energy expenditure, fat oxidation and body weight, reported by 134 RCTs. The effects ranged the full spectrum, from negative to null and positive effects, for each outcome. Drinking water reportedly lowers, has no effect, and increases energy intake, energy expenditure, fat oxidation and weight.

Countries and communities worldwide are promoting drinking water for weight management, implicitly expecting drinking water to lower energy intake, increase energy expenditure, increase fat oxidation, decrease weight gain (support obesity prevention) and/or facilitate weight loss (support obesity treatment). This expectation may not be met if the range of possible effects of drinking water is not restricted to the desired effects. Indeed, of 115 reported effects of drinking water on weight from RCTs, 83 were null effects. Meta-analyses of beverage change effects on weight report small average effect sizes across studies and conclude that evidence is inconsistent and weak [19,42]. If the studies test fundamentally different conditions and questions, however, it may be misleading to estimate an average effect size across studies [43,134].

Mattes *et al.* [43] call for work to resolve the discrepant literature and “move the field forward”. They argue that clinical recommendations and public policy should be better designed to meet the needs of the susceptible group(s), given that beverage effects may not be effective for everyone, and one-size-fits-all recommendations and policy may place undue restrictions on the total population. To inform such better design, studies are needed to characterize the underlying causal mechanism(s) [43].

To specify drinking water interventions, to restrict the range of possible effects to the desired effects, public health policy makers and practitioners need to be able to define the “active ingredient”, which may include aspects of the background context [135]. The setting in which the intervention is delivered and/or elements of the intervention process influence the intervention outcome and generalizability. “Any attempts to extrapolate from study settings to the real world are hampered by a lack of understanding of the key elements of individuals and the settings in which they were trialed” [136].

The findings of this review suggest that key contextual elements for drinking water effects on weight may include the types of beverages consumed at baseline, *i.e.*, the types of beverages to potentially be replaced, individual dietary restraint, food preferences, background diet, activity level, weight status and “metabolic flexibility”. Given the potential for negative, null or positive effects of drinking water on energy intake, as Andrade *et al.* [67] observe, “recommendations directed at changing eating behavior for the purpose of energy intake reduction should be made carefully.”

### 4.1. Aligning Conditions Associated with Short- and Long-Term Effects of Drinking Water

#### 4.1.1. Conditions Associated with Null Effects

In short-term RCTs, null effects of drinking water on meal energy intake are observed when drinking water does not replace caloric beverages or is consumed instead of “diet” drinks with *ad libitum* food, offered instead of a caloric beverage to restrained eaters, or offered with non-preferred foods to young children. Drinking water has null effects on energy expenditure for normal weight individuals who can alter fuel partitioning instead of energy expenditure. Drinking water does not increase fat oxidation during high-intensity exercise, when anaerobic metabolism and carbohydrate oxidation predominate [137]. Drinking water does not increase fat oxidation when high glycemic foods are consumed under resting conditions, because increases in blood glucose and insulin suppress fat oxidation. When high glycemic foods are consumed, postprandial insulin concentrations do not vary significantly by beverage type [115]. Drinking water does not increase fat oxidation when the volume of drinking water consumed is less than 0.5 L and/or there is an indication of cell dehydration (elevated serum or urine osmolality), under fasting conditions, at rest or during low-to moderate-intensity exercise.

In longer term RCTs, conditions associated with null effects on weight change are similar to those associated with short-term null effects. Longer term RCTs report no effect of drinking water on weight change when not all caloric beverages are targeted for replacement/displacement, when the control or reference condition for relative drinking water effects is not a caloric beverage, when there is a low baseline intake of SSB, when caloric beverages do not change as a result of the intervention, when participants have restricted food intake, and when the volume of drinking water is not enough to dilute urine. It is concerning that recommendations to drink water for weight management in the United States (see Table 1) advocate conditions that are associated with a null effect on weight change in the literature.

#### 4.1.2. Conditions Associated with Less Weight Gain or More Weight Loss

Table 7 summarizes RCT conditions of studies that report that drinking water lowers energy intake, increases energy expenditure and/or increases fat oxidation. Drinking water lowers energy intake and increases fat oxidation when it is consumed instead of all caloric beverages, not only soda, but also milks, juices and alcohol, by non-dieting individuals, regardless of age, sex and weight status, with *ad libitum* food intake and either resting or low- to moderate-intensity exercise.

Drinking water increases energy expenditure for obese individuals who are fasting and sitting at rest, if they consume 0.5 L or more and have no change in RQ or heart rate. Drinking water increases fat oxidation for individuals who are fasting (or have fasting glucose and insulin concentrations), at rest or during low- to moderate-intensity exercise, if the volume consumed is 0.5 L or more.

**Table 7:** RCT conditions where short-term effects of drinking water favor weight management. RQ, respiratory quotient.

Effect of Drinking Water	Conditions for Effect	Type of Drinking Water Exposure
Lower energy intake	*Ad libitum* food	Drinking water *vs.* caloric beverages (not only sugar-sweetened beverages)
Greater fat oxidation	At rest or LM exercise Lower insulin	Drinking water *vs.* caloric beverages (not only sugar-sweetened beverages)
Greater energy expenditure	Obese individuals Fasting At rest Volume ≥500 mL No change in RQ or heart rate	Drinking water *vs.* no fluid
Greater fat oxidation	Fasting At rest or LM exercise Volume ≥500 mL Lower insulin	Drinking water *vs.* no fluid

LM, low- to moderate-intensity exercise.

Table 8 summarizes the effects of drinking water on weight outcomes, stratified by type of drinking water exposure and RCT conditions. Drinking water results in less weight gain for individuals with *ad libitum* diet and usual exercise, when it is consumed instead of all caloric beverages, not only SSB. Drinking water results in greater weight loss for overweight or obese individuals when diet is restricted, the usual level of exercise is maintained and the volume of drinking water exceeds 1 L/day, enough to dilute urine.

**Table 8:** Long-term effects of drinking water on weight by type of exposure and RCT conditions.

Type of Drinking Water Exposure	Conditions for Effect	Effect of Drinking Water
Drinking water *vs.* sugar-sweetened beverages only	*Ad libitum* food Usual exercise All individuals	No effect
Drinking water *vs.* caloric beverages	Restricted food	No effect
Drinking water *vs.* caloric beverages	*Ad libitum* food Usual exercise All individuals	Less weight gain
Volume >1+ L/day *vs.* <1 L/day or dilute *vs.* concentrated urine	Hypocaloric or low glycemic diet Lower insulin Usual exercise Overweight or obese individuals	Greater weight loss

### 4.2. Hypotheses to Pursue in Future RCTs and Meta-Analyses

The results of this review suggest that exposures and conditions that might optimize drinking water effects for weight management may include:

(1) Drinking water instead of all caloric beverages for non-dieting individuals who are consuming low-glycemic, low-fat food *ad libitum* (not deliberately or consciously restricting food intake) and maintaining usual levels of low- to moderate-intensity physical activity: These conditions would be hypothesized to prevent weight gain via decreases in beverage and total energy intake and increases in fat oxidation that allow individuals to burn up the fat consumed in each meal before their next meal, even if they sit at rest during the postprandial period [40]. When drinking water is consumed instead of a caloric beverage with low glycemic foods, the postprandial rise in serum insulin is significantly smaller and of shorter duration (< 1 h *vs.* 2 h) [115].
(2) Increasing drinking water by more than 1 L/day for overweight or obese individuals with a restricted, hypocaloric and/or low glycemic diet that lowers blood glucose and insulin levels: The latter conditions would be hypothesized to accelerate weight loss by increasing energy expenditure and/or fat oxidation by reducing osmotic stress on cells, improving insulin resistance, reducing gluconeogenesis and/or improving postprandial glucose clearance. Because the caloric deficit is pre-defined and consciously maintained in this condition, the effects of drinking water on weight loss observed under this condition would not be expected to be mediated by a change in total energy intake. The following states favor fat oxidation: fasting, cell hydrated, not oxygen-limited, low cortisol/stress, moderate- and low-intensity exercise.

Intake of high glycemic, starchy staple food at each meal can be expected to suppress fat oxidation during the postprandial period and mask drinking water effects. Multi-component interventions have tested drinking water effects concurrent with shifts in fruit and vegetable and/or fat intake, but have yet to test for interactive effects of drinking water (beverage with zero glycemic, zero insulinemic index) and low glycemic food intake (glycemic index <50, glycemic load <11).

The hypothesized mechanisms can also be expected to be subject to effect modification by factors such as age, sex, body size, fat intake, exercise, insulin levels and metabolic flexibility or adaptation. Very young children may be more able to compensate for beverage calories and, thus, less able to benefit from drinking water in terms of lower total energy intake. Young males and obese individuals are more likely to have elevated urine osmolality (evidence of osmotic stress on cells) [138,139,140] and lower fat oxidation rates [141,142] and may require a greater absolute dose of drinking water to dilute urine and observe an effect on weight than girls and normal weight individuals. High fat intake may cause a positive fat balance, despite a significantly increased rate of fat oxidation. High-intensity exercise favors carbohydrate oxidation over fat oxidation, regardless of the type of beverage consumed. Individuals may alter energy intake, energy expenditure and/or fat oxidation at later meals or on subsequent days to compensate for the acute effects of drinking water (e.g., [82,143]). To determine if and/or how RCT conditions can be leveraged to maximize the impact of drinking water interventions for weight management, studies are needed to clarify causal mechanisms, including effect modifiers.

Contrary to assertions that “targeting water and diet drinks may not be important or necessary in the interventions aimed at reducing the consumption of sugar sweetened beverages among adolescents [144]”, explicitly specifying the target beverage change in absolute and relative terms may help to optimize intervention effects. Drinking water effects are contingent on other beverage intake and/or food intake, which can shift deliberately or unconsciously. Even with no change in absolute intake of drinking water, the relative intake of drinking water changes when other beverage intake is increased or decreased. In the study by Veitch *et al.* [144], for example, while absolute intake of drinking water remained constant, relative intake of drinking water increased from 26% to 38% of beverage intake. Relative shifts in the beverage intake pattern significantly impact the composition and osmolality of beverage intake, which in turn impact beverage absorption and metabolism. *A priori* specification of target absolute and relative beverage and food intake may avoid wasted investment from unanticipated beverage and food shifts. 

The RCT effects reviewed here suggest that beverages sweetened with non-caloric sweeteners, aspartame, acesulfame-K, sucralose and/or saccharin have comparable effects to drinking water with respect to energy intake. Further research is needed to determine if non-caloric sweeteners are comparable to drinking water with respect to effects on energy expenditure, fat oxidation and weight change, accounting for *ad libitum* or restricted diet and beverage volume.

Accumulated data from crossover experiments and RCTs indicate that milk, even skim milk, is not comparable to drinking water in terms of its effects on energy intake, fat oxidation and weight change. Milk results in significantly greater energy intake, less fat oxidation and more weight gain [130] than drinking water. Milk is proposed as an alternative to drinking water, because of its nutritional value (see Table 1), concern that individuals will fail to meet nutrient intake recommendations if they eliminate milk from the diet [145] and the belief that milk facilitates weight management [130]. It is important to clarify that milk results in greater weight loss when it replaces calories from food [146], not when it replaces drinking water in the diet [130]. RCTs are needed to test the weight management effects of drinking water instead of other beverages and eating dairy instead of other food.

### 4.3. Limitations

Several factors limit the interpretation of the present results. The goal of this exploratory review was to identify conditions associated with different types of drinking water effects. The goal was not to estimate a generalizable, average effect size across studies. This qualitative analysis draws on a set of unweighted, non-independent effects of drinking water from heterogeneous RCTs for hypothesis generation purposes only. It remains to be determined if the conditions that appear to be associated with particular effects, in this analysis, in fact cause negative, null and/or beneficial outcomes of drinking water for particular population groups, in controlled trials and quantitative meta-analyses. Effective drinking water recommendations and interventions depend on clarifying the causal mechanism(s) of effect(s).

The RCTs included in this review may not represent all RCTs reporting the effects of drinking water on the outcomes of interest, because RCTs not published in English and/or not indexed in PubMed may have been missed. All references in published systematic reviews and meta-analyses summarizing drinking water effects of weight change were, nevertheless, considered for inclusion in this analysis. Previous reviews apply stringent inclusion/exclusion criteria, following PRISMA guidelines (e.g., [19]).

The review excludes effects of drinking water over periods longer than 24 h, but shorter than one month, so does not contribute information about compensation over hours or days that might negate or magnify short-term effects of drinking water. The review did not consider conditions associated with the effects of drinking water exposures sustained between two days and one month. Chronic exposure to drinking water, over multiple days or weeks, can result in metabolic and physiologic changes not seen with acute exposure to drinking water [147]. The alignment of conditions associated with beneficial effects of drinking water over <12 h and >1 month, nevertheless, suggests that short-term effects of drinking water do translate, at least to a certain extent, into long-term weight change.

The review only systematically abstracted information about participant age, gender, weight status, diet and activity. Future studies should also consider other key effect modifiers and elements of context not covered in this study, such as the timing of the drinking water exposure, participant health and metabolic flexibility, in particular. The timing of beverage intake relative to meals may modify beverage effects (e.g., [67,68,143]). More research is needed to determine if overnight water-restricted older, overweight or obese individuals can lower energy intake by drinking water before meals, and if normal weight individuals might lower total energy intake by drinking more water between mouthfuls during meals.

Limited information was available from RCTs about the relative adequacy of water intake (e.g., total water intake, hydration biomarkers), diet and activity. It is unknown if unspecified usual dietary intake involves high glycemic foods, energy-dense (low water) foods and/or typical Western diets and/or if the unspecified level of usual activity is sedentary. Studies are warranted to describe diet and activity conditions in greater detail.

Further work is needed to corroborate associations between RCT conditions and the direction of effect, accounting for parameters of study quality, which were not addressed here. RCTs were included regardless of sample size, participant health status, study quality, protocol deviations, non-adherence, loss to follow-up and stratified and no intention to treat analyses. Intervention targets not met are frequent in longer term RCTs (e.g., [29,129,148]).

More effort is needed to align short- and long-term effects of drinking water in a focused way for particular target populations and conditions. Doing this will require filling gaps in the short- and long-term literature. For example, to align and inform interventions for non-athlete, overweight and obese children, short-term RCTs and meta-analyses are needed to characterize short-term effects of drinking water on energy intake, energy expenditure and fat oxidation in non-athlete, overweight or obese children, sitting at rest.

Studies published after December 2014 were not included in the present analysis. One 12-week RCT, published after that date [149], reports that obese adults randomly assigned to drink 500 mL water 30 min before their main meals lost significantly more weight than the control group, assigned to imagine their stomach was full before meals. The results are consistent with the weight loss effects of increasing drinking water by more than 1 L/day for overweight or obese individuals with a restricted, hypocaloric or low glycemic diet [25,129,131].

## 5. Conclusions

RCTs report negative, null and positive effects of drinking water on energy intake, energy expenditure, fat oxidation and weight change. Heterogeneity across studies is associated with different study conditions. Conditions that are associated with null effects over the short term are also associated with null effects over the longer term. To optimize drinking water initiatives for weight management, future research, meta-analyses and public health interventions should pursue conditions that decrease energy intake, increase energy expenditure and/or increase fat oxidation.

## Supplementary Materials

Supplementary materials can be accessed at: http://www.mdpi.com/2072-6643/8/1/19/s1.
